# Mortality risk prediction model in AIDS patients with pneumocystis pneumonia in China

**DOI:** 10.3389/fcimb.2024.1485231

**Published:** 2025-02-07

**Authors:** Xi Wang, Letian Liu, Wen Wang, Yang Zhang, Hui Chen, Zhangli Wang, Jianwei Li, Yue Gao, Yanqun Huang, Lijun Sun, Tong Zhang, Aixin Li

**Affiliations:** ^1^ Center for Infectious Diseases, Beijing Youan Hospital, Capital Medical University, Beijing, China; ^2^ School of Biomedical Engineering, Capital Medical University, Beijing, China

**Keywords:** HIV/AIDS, pneumocystis pneumonia, mortality, prediction model, prognosis, risk factor

## Abstract

**Introduction:**

Pneumocystis pneumonia (PCP) is a common and serious complication of HIV/AIDS, with a higher prevalence in patients not receiving antiretroviral therapy. Due to the high mortality rate of PCP, accurate prediction of its case fatality rate is very important for clinical treatment. We aimed to develop a risk model for the near-term prognosis of people with HIV/AIDS and PCP and verify its effectiveness.

**Methods:**

This single-center, retrospective observational study was conducted at Beijing Youan Hospital from January 2012 to October 2022. 972 AIDS patients with Pneumocystis pneumonia met our criteria were recruited. The patients were divided into death group and survival group according to clinical outcome during hospitalization. Data of the two groups were collected including general information and laboratory test results. 53 medical characteristics of the two groups were collected. Prediction variables were screened with Multivariate logistic regression analysis and Lasso regression model. We used ROC curve to identify the discrimination of training and testing data sets. The Shapley Additive exPlanation (SHAP) method was applied to explain the final model and the weights of features.

**Results:**

The overall mortality rate among hospitalized patients was 17.8%. We found that the best prediction effect can be obtained when ALB, PO_2_, TBIL, LDH, CD4^+^ T lymphocyte counts are incorporated into the PCP risk prediction model. The model had a perfect discrimination with AUC of 0.994 and 0.947 in training and validation cohorts. The prognosis risk grade was divided into three grades: low-risk group (0-25 points with mortality of 5.9%), moderate-risk group (25-50 points with mortality of 45.1%) and high-risk group (above 50 points with mortality of 80%). There is a statistically significant difference in mortality among these three grades (χ^2^ = 419.271, P<0.001).

**Conclusion:**

We developed and validated a model of the prognostic risk level of PCP in patients of AIDS with the results of blood tests reviewed by patients at routine visits. The model is more convenient to use, allowing clinicians to obtain a determined probability value of PCP mortality with simple calculations within the first 72 hours of the patient’s admission.

## 1 Introduction

Pneumocystis pneumonia (PCP) is an opportunistic respiratory infection caused by Pneumocystis, and it serves as an indicative disease of HIV/AIDS, representing the most severe and prevalent pulmonary complication as well as a leading cause of mortality among HIV/AIDS patients. Since the 1980s, the incidence of PCP has increased rapidly, with approximately 70% to 80% of AIDS patients co-infected with PCP. After the advent of ART, the current incidence of PCP among HIV-infected individuals in China is 8% to 26% (AIDS and Hepatitis C Professional Group, Society of Infectious Diseases, Chinese Medical Association, 2024). Before the advent of antiretroviral therapy, the death rate of PCP ranged from 10% to 30% (Benson et al., 1991; Bennett et al., 1994), with severe cases reaching up to 35% to 85% (De Palo et al., 1995; Bédos et al., 1999). In the era of highly active anti-retroviral therapy (HAART), the death rate of PCP can still be as high as 35% (Kann et al., 2021). This underscores the importance for infectious disease physicians to address the challenge of predicting the case fatality rate of PCP. Therefore, it is crucial to identify and validate relevant risk factors associated with PCP mortality. Predicting mortality rates helps optimize medical resource allocation, improve patient and family compliance, enable early intervention, ensure high-risk patients receive better treatments, and enhance long-term outcomes.

Previous retrospective studies have identified several possible risk factors, including hemoglobin (HGB), arterial oxygen pressure (PaO_2_), old age, Kaposi’s sarcoma of the lung, etc (Fei et al., 2009; Wang et al., 2021). However, most of these studies are based on data from western countries. In China, there is a lack of established predictive scoring models for predicting the prognosis of HIV/AIDS-related PCP. Therefore, we conducted a retrospective observational cohort study to determine the objective and independent predictors of death available at the time of disease onset, deduce the PCP mortality prediction rules, and stratify patients based on their risk of inpatient mortality, so as to provide scientific basis for subsequent clinical treatment.

## 2 Materials and methods

### 2.1 Study design

This is a single-center retrospective observational study conducted at Beijing Youan Hospital, Capital Medical University. It has been registered in China Clinical Trial Registry (ChiCTR2100048218). The study was in accordance with the Declaration of Helsinki (revised in 2013) and was approved by Ethics Committee of Youan Hospital, Capital Medical University (LL-2021-002-K), informed consent was waived. All clinical assessments were performed according to the follow-up requirements of our treatment center. No additional testing or examination is required for this study. Data were collected by having access to an electronic medical record chart.

### 2.2 Study population and diagnosis

Patients were recruited in our study who were diagnosed as HIV/AIDS with PCP and hospitalized in Beijing Youan Hospital, Capital Medical University from January 2012 to October 2022. The inclusion criteria were as follows: 1) age ≥18 years, 2) The diagnostic criteria for HIV/AIDS and PCP refer to the diagnostic criteria in the Chinese Guidelines for Diagnosis and Treatment of HIV/AIDS (2021 Edition) issued by the HIV/AIDS and Hepatitis C Professional Group, Society of the Infectious Diseases, Chinese Medical Association and the Chinese Center for Disease Control and Prevention in 2021 (Acquired Immunodeficiency Syndrome and Hepatitis C Professional Group, Society of Infectious Diseases, Chinese Medical Association, Chinese Center for Disease Control and Prevention, 2022). All patients were confirmed with anti-HIV-1 antibody screening by enzyme-linked immunosorbent assay (ELISA) and Western blotting test (WB). The diagnostic criteria of PCP were: (1) subacute onset, dyspnea gradually aggravated, accompanied by fever, dry cough, chest tightness, symptoms gradually worsening, and severe respiratory distress; (2) There are few positive signs in the lung, or a few scattered dry and wet rales can be heard. The severity of physical signs and disease symptoms is often out of proportion; (3) Chest X-ray examination showed diffuse reticular nodular interstitial infiltration in both lungs starting from the pulmonary hilum. Lung computerized tomography (CT) examination showed ground glass changes in both lungs. 13%~18% of the patients were accompanied by bacterial or mycobacterial infections, and pulmonary imaging could have corresponding pulmonary imaging manifestations; (4) Blood gas analysis indicated hypoxemia with significant decrease in PaO_2_ levels often falling below 60mmHg (1mmHg = 0.133kPa); (5) Blood lactate dehydrogenase (LDH) is often > 500mg/dl; Plasma (1,3)- β-D-glucan (BDG) levels were significantly higher than normal values; (6) The diagnosis depends on the detection of cysts or trophozoites of pneumocystis by pathogenic examination such as sputum or bronchoalveolar lavage/lung biopsy. Polymerase chain reaction (PCR) is also an alternative diagnostic method.

The exclusion criteria were: the patients lacking baseline data and those with unobservable clinical outcomes.

### 2.3 Treatment

According to the diagnosis, treatment and prevention of common opportunistic infections in Chinese Guidelines for Diagnosis and Treatment of HIV/AIDS (2021 Edition) (Acquired Immunodeficiency Syndrome and Hepatitis C Professional Group, Society of Infectious Diseases, Chinese Medical Association, Chinese Center for Disease Control and Prevention, 2022), trimethoprim/sulfamethoxazole (TMP-SMZ) was given orally at a dosage of sulfamethoxazole 400mg and trimethoprim 80mg, 4 tablets per dose, 3 times a day. AIDS patients with moderate-to-severe PCP, defined as alveolar arterial oxygen partial pressure difference (P(A-a)O_2_) > 35mmHg or PaO_2_ < 70mmHg were also given intravenous drip of methylprednisolone, 80mg/d for 1-5 days, followed by 40mg/d for 6-10 days, and then reduced to 20mg/d for the remaining days up to a total course of treatment lasting for 21 days. Among them, 75 patients were treated with caspofen at a dosage of 70mg/d initially and then reduced to50 mg/d for a period ranging from3-14 days. Etiological treatment should be accompanied by oxygen therapy and management of complications.

Some patients have started ART before admission, while others have started ART as soon as their conditions permit.

### 2.4 Data collection and classification

The main outcome of this study is whether there is death during hospitalization. The prediction of death is coded as a binary dependent variable categorized as 0 (the patient did not die during hospitalization) and 1 (the patient died during hospitalization). To predict the likelihood of patient mortality during hospitalization, we obtained demographic information (gender, age, route of infection), medical history (course of disease, HIV infection time), symptoms (fever, cough, shortness of breath), important vital signs (respiration, heart rate), laboratory test results within 72 hours after admission (arterial blood gas analysis, complete blood count, C-reactive protein [CRP], liver and kidney function tests, electrolyte levels, lactate dehydrogenase [LDH], beta-D-glucan [BDG], CD4 T lymphocyte count), antiretroviral therapeutic regimen, complications and co-infection from clinical medical record system.

Patients were divided into training and testing cohorts according to the date of admission. A prognostic model was developed with data from January 2012 to December 2019 for the training cohort and validated with data from January 2020 to October 2022 for the testing cohort.

### 2.5 Statistical analysis

Continuous variables were expressed as the mean with standard deviation (SD), and the Kolmogorov-Smirnov test was used to test the normal distribution. Independent t-test were performed if the variables were normally distributed, otherwise Mann-Whitney U test would be adopted. Categorical variables were expressed as frequencies with percentages and analyzed by chi-square test or Fisher’s exact test when necessary.

In terms of variable screening, we utilized the least absolute shrinkage and selection operator (LASSO) in addition to multivariate logistic regression. Lasso is a method for simultaneous feature selection and regularization by adding an L1 norm penalty to the calculation of minimum residual sum of squares (RSS). Tuning parameter λ (0.351) of 1 standard error to the minimum was determined through cross-validation in this study.

A prognosis model was established with patient data from January 2012 to December 2019, and was validated with data from January 2020 to October 2022. Model performance was assessed using area under the receiver operating curve (ROC) curve (AUC), accuracy, precision, recall, and F1-score. The prediction under the dichotomy problem can result in true positive (TP), true negative (TN), false positive (FP) and false negative (FN). The evaluation indicators are calculated as follows:


Accuracy =(TP+TN)/( TP+TN+FP+FN);

Precision = TP /(TP +FP);

Recall = TP /(TP + FN);

F1−score = 2 Precision × Recall /(Precision +Recall)

The AUC is the area under the operating characteristic curve, reflecting the relationship between sensitivity and specificity. The AUC value is significant for model selection and decision making, with values < 0.50 indicating no predictive power, 0.50 - 0.70 moderate accuracy, 0.71 - 0.90 moderate to high accuracy, and > 0.90 high accuracy.

Calibration curve analysis was applied to assess the accuracy of the risk prediction of the prognostic model, that is, the agreement between the predicted probability and the actual observed probability in HIV/AIDS patients with PCP. The Hosmer-Lemeshow goodness-of-fit test was performed on the prognostic model, and P>0.05 indicated statistically insignificant difference and good model calibration performance.

Decision curve analysis (DCA) was performed to validate the established prognostic model for clinical application. DCA is utilized to investigate the impact of the prognostic model on the net clinical benefit rate at various positive thresholds to ascertain its performance in clinical settings. The x-axis of the graph represents the Threshold Probability (TP). The probability of death for patient is denoted as Pi when the prognostic model assessment reaches a certain value; it is considered positive when Pi reaches a specific threshold value (denoted as Pt). The implementation of a particular intervention naturally shifts the balance between benefits and harms related to patient survival and mortality. The y-axis represents the Net Benefit (NB) after subtracting harm from benefit, and the DCA curve is obtained by plotting NB against Pt.

In this study, Sharpley Additive exPlanations (SHAP) values were employed as the feature weights to interpret the output of a machine learning model. SHAP is a Python package for model interpretation based on game theory. It approximates a trained prediction model with a simpler model to calculate the contribution of each feature in the form of a SHAP value, allowing for additive feature attribution (Lundberg and Lee, 2017). Positive or negative SHAP values reflect the influence on the prediction, and a feature’s importance is computed as the average of its absolute SHAP values across all samples. For local interpretability, each feature has its own set of SHAP values, enabling clinicians to analyze the reliability of the prediction model for each sample. Global interpretation is obtained by averaging the SHAP values across all samples for each variable. In this paper, the correlation between each characteristic and the risk of death in HIV/AIDS patients with PCP was analyzed by calculating the SHAP value of each clinical indicator and the model interpretation was performed by combining the results of relevant clinical studies and clinical reality.

We utilized univariate analysis and multivariate logistic regression analysis from the statsmodels.api package for variable screening and LASSO regression for feature screening and logistic regression prediction model building and validation with the sklearn package. The calculation of SHAP values and plotting of images were conducted with the SHAP package in Python while curve plotting for decision curve analysis and calibration curve analysis was with python software.

## 3 Results

### 3.1 Study population

**Figure 1:**
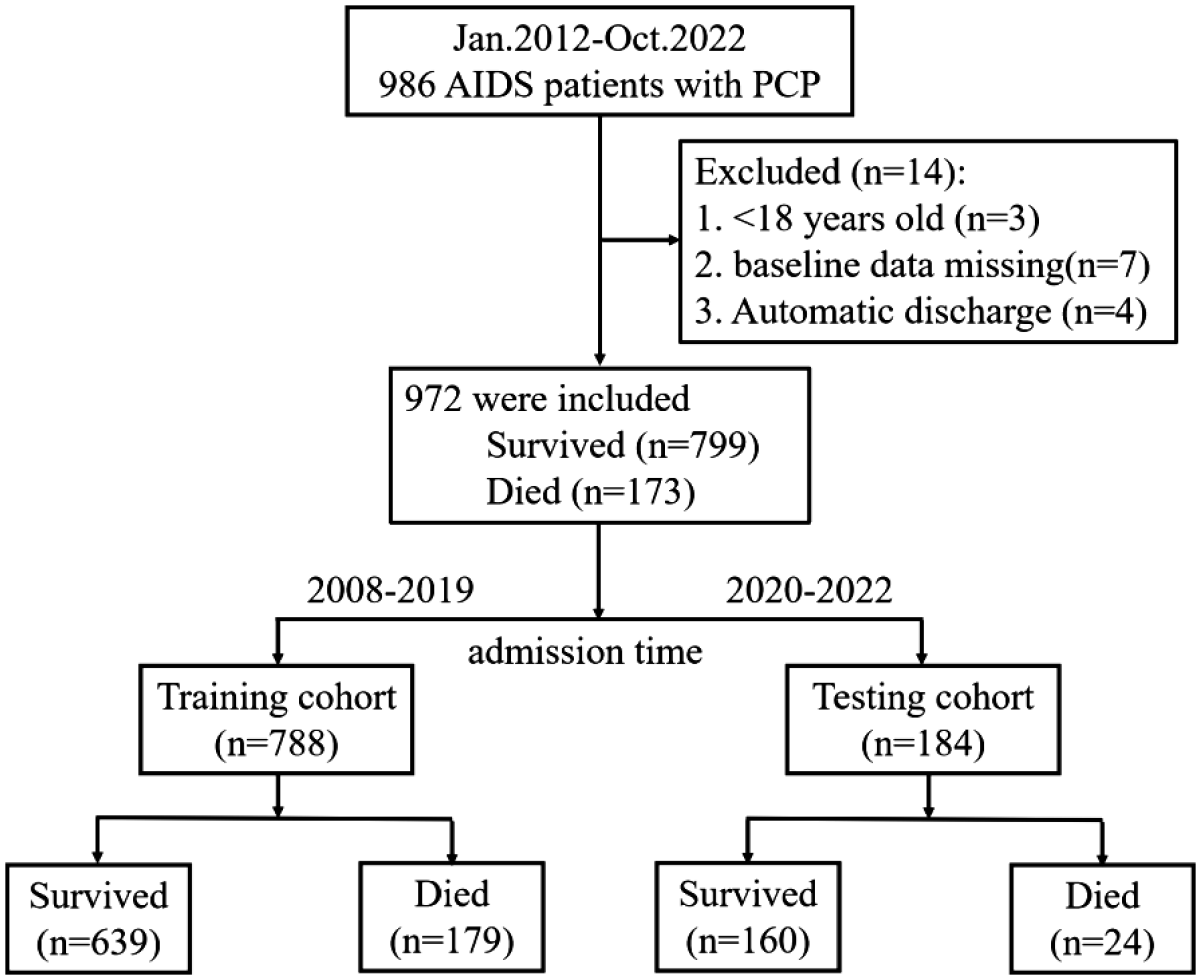
Patient flow chart. The process of screening and selecting patients who were in line with our inclusion criteria and flow chart of allocation according to admission time.

From January 2012 to October 2022, a total of 986 patients were hospitalized in Beijing Youan Hospital, Capital Medical University. After excluding patients who did not meet our criteria, 972 patients were finally included in our study, of which 173 (17.8%) died during hospitalization. Flow charts of patient inclusion and prognosis were shown in **Figure 1**.

### 3.2 Clinical characteristics

**Table 1** summarizes the characteristics of HIV/AIDS patients with PCP (n = 972). The mean age of the 972 cases at baseline was 38.00 (30.00, 48.00) years, with the majority being male (93.9%). Univariate analysis revealed that the clinical features that were significantly related to the death of HIV/AIDS patients with PCP during hospitalization. These included age (39.2-40.7 years, P=0.001), heart rate (91.7-92.3 bpm, P<0.001), respiratory rate (RR)(21.7-22.2 bpm, P<0.001). P(A-a)O_2_ (37.8-40.0 mmHg, P<0.001), white blood cell (WBC) (5.5-5.9×10^9/L, P=0.001), neutrophils (N) (4.2-4.6×10^9/L, P<0.001), aspartate aminotransferase (AST) (47.7-57.5 U/L, P<0.001), total bilirubin (TBIL) (9.1-10.5 U/L, P<0.001), direct bilirubin (DBIL) (3.5-4.4 U/L, P<0.001), blood urea nitrogen (BUN) (4.7-5.0 mmol/L, P<0.001), lactate dehydrogenase (LDH) (393.9-353.2 U/L, P<0.001) and C-reactive protein (CRP) (49.7-55.9 mg/L, P<0.001) were significantly higher in the patients of the death group than of the survival group. In contrast, patients in the death group had partial pressure of carbon dioxide (PCO_2)_ (34.5-32.3 mmHg, P=0.014), PaO_2_ (70.3-72.3 mmHg, P<0.001), oxygen saturation (SPO_2)_ (91.9-92.7%, P<0.001), red blood cell (RBC) (3.8-4.0×10^12/L, P=0.003), HGB (111.8-114.4 g/L, P<0.001), platelet (PLT) (215.5-228.3×10^9/L, P<0.001), lymphocyte (L) (0.8-0.9×10^9/L, P<0.001), albumin (ALB) (30.6-31.2 g/L, P<0.001), creatinine (CRE) (64.2-70.4 μmol/L, P=0.004), cholesterol (CHO) (3.5-3.6 mmol/L, P= 0.004), prealbumin (PALB) (166.1-189.3 mg/L, P<0.001), cholinesterase (CHE) (4990.9-5238.4 U/L, P<0.001), sodium (Na) (134.3-135.0 mmol/L, P<0.001), chlorine (Cl) levels (99.2-99.8 mmol/L, P<0.001), CD4+ cell count (39.8-47.4 cell/μL, P<0.001) and CD4/CD8 ratio (0.0900-0.1069, P<0.001) were significantly lower than those of the survival group. Co-infection with bacteria (P=0.003), tuberculosis (TB) (P=0.031), cryptococcus (P=0.002), cytomegalovirus (CMV) (P=0.033) and pneumothorax (P<0.001) were also associated with a worse prognosis in HIV/AIDS patients with PCP.

**Table 1:** Characteristics of total patients.

Variables	Total (n = 972)	Survived (n = 799)	Died (n = 173)	*P*
Demographics				
Age (years)	38.00 (30.00,48.00)	38.00 (30.00,48.00)	41.00 (34.00,50.00)	0.001
Gender (M/F)	913/59	749/50	164/19	0.725
Course of disease (d)	21.00 (10.00,37.50)	21.00 (10.00,40.00)	20.00 (10.50,30.00)	0.943
HIV infection time (d)	14.00 (3.00,202.50)	14.00 (3.00,180.00)	14.00 (2.00,365.00)	0.847
Transmission route				0.178
Homosexual	761 (78.29%)	630 (78.80%)	131 (75.70%)	
Heterosexual	172 (17.70%)	135 (16.90%)	37 (21.40%)	
Blood transfusion	31 (3.19%)	26 (3.30%)	5 (2.90%)	
Intravenous drug	8 (0.82%)	8 (1.00%)	0 (0.00%)	
ART-naive	830 (85.39%)	682 (85.36%)	148 (85.55%)	1.000
Vital signs				
HR (bpm)	92.00 (82.00,106.00)	90.00 (82.00,104.00)	100.00 (88.00,111.00)	<0.001
RR (bpm)	21.00 (20.00,23.00)	20.00 (20.00,22.00)	22.00 (20.00,26.00)	<0.001
Symptom				
Fever	805 (82.82%)	662 (82.85%)	143 (82.66%)	1.000
Cough	682 (70.16%)	569 (71.21%)	113 (65.32%)	0.148
Shortness of breath	706 (72.63%)	573 (71.71%)	133 (76.88%)	0.198
Laboratory test				
PH	7.43 (7.41,7.45)	7.43 (7.41,7.45)	7.43 (7.41,7.46)	0.218
PCO_2_ (mmHg)	31.10 (28.00,35.10)	31.40 (28.30,35.10)	29.50 (26.30,34.60)	0.014
PO_2_ (mmHg)	70.85 (58.25,85.53)	76.20 (64.90,87.60)	53.10 (46.98,59.58)	<0.001
P (A-a)O_2_ (mmHg)	39.00 (24.10,54.10)	35.30 (21.70,47.10)	59.20 (51.45,66.35)	<0.001
SpO_2_ (%)	94.40 (90.20,97.00)	95.50 (92.50,97.30)	86.15 (78.53,90.80)	<0.001
WBC (×10^9/L)	5.21 (3.60,7.19)	5.03 (3.60,6.90)	5.91 (3.59,9.15)	0.001
RBC (×10^12/L)	3.91 (3.38,4.36)	3.93 (3.44,4.38)	3.75 (3.06,4.27)	0.003
HGB (g/L)	114.50 (100.00,128.00)	116.00 (102.00,128.00)	109.00 (90.00,125.00)	<0.001
PLT (×10^9/L)	218.00 (152.25,283.75)	223.00 (163.00,289.00)	181.00 (98.50,265.50)	<0.001
L (×10^9/L)	0.69 (0.43,1.06)	0.74 (0.48,1.11)	0.41 (0.26,0.75)	<0.001
N (×10^9/L)	3.76 (2.38,5.76)	3.54 (2.30,5.27)	5.07 (3.04,7.83)	<0.001
ALT (U/L)	29.25 (17.10,52.28)	28.90 (16.80,53.40)	31.20 (20.65,48.05)	0.538
AST (U/L)	34.55 (24.20,53.88)	32.80 (23.60,51.30)	43.30 (32.10,74.15)	<0.001
TBIL (μmol/L)	7.90 (6.10,10.90)	7.70 (5.90,10.00)	10.30 (7.25,14.70)	<0.001
DBIL (μmol/L)	2.60 (1.70,3.90)	2.50 (1.60,3.70)	3.40 (2.10,6.00)	<0.001
ALB (g/L)	31.20 (27.30,34.60)	32.30 (29.30,35.20)	24.30 (21.35,26.35)	<0.001
BUN (mmol/L)	4.27 (3.30,5.63)	4.10 (3.24,5.30)	5.22 (4.09,6.85)	<0.001
CRE (μmol/L)	63.45 (53.53,73.08)	63.90 (55.00,73.40)	60.20 (47.30,71.90)	0.004
CHO (mmol/L)	3.48 (2.92,4.13)	3.52 (2.96,4.13)	3.21 (2.65,4.01)	0.004
PALB (mg/L)	153.75 (102.10,221.93)	162.00 (110.70,236.70)	104.70 (69.80,148.10)	<0.001
CHE (U/L)	5102.50 (3720.00,6395.75)	5439.00 (4190.00,6706.00)	3353.00 (2319.00,4460.00)	<0.001
K (mmol/L)	3.97 (3.61,4.35)	3.98 (3.61,4.35)	3.90 (3.55,4.32)	0.470
Na (mmol/L)	135.10 (132.20,137.60)	135.40 (132.60,137.90)	133.30 (129.20,136.20)	<0.001
Cl (mmol/L)	99.80 (96.63,102.48)	100.00 (97.10,102.50)	98.50 (94.80,101.65)	<0.001
LDH (U/L)	344.20 (252.10,486.20)	322.70 (245.15,426.83)	525.30 (409.55,720.50)	<0.001
BDG (pg/mL)	100.70 (10.00,278.60)	104.75 (14.83,278.75)	88.90 (10.00,277.20)	0.313
CD4 (cell/μL)	22.00 (9.00,48.75)	26.00 (10.00,58.00)	10.00 (4.00,24.25)	<0.001
CD4/CD8	0.06 (0.03,0.11)	0.06 (0.03,0.12)	0.04 (0.02,0.09)	<0.001
CRP (mg/L)	33.70 (12.00,80.00)	30.00 (11.00,69.00)	64.00 (24.00,113.00)	<0.001
Comorbidities				
Bacterial pneumonitis	743 (76.44%)	595 (74.47%)	148 (85.55%)	0.003
Tuberculosis	275 (28.29%)	214 (26.78%)	61 (35.26%)	0.031
Candida	405 (41.67%)	330 (41.30%)	75 (43.35%)	0.681
Cryptococcus	26 (2.67%)	15 (1.88%)	11 (6.36%)	0.002
Penicillium marneffei	11 (1.13%)	7 (0.88%)	4 (2.31%)	0.221
CMV	188 (19.34%)	144 (18.02%)	44 (25.43%)	0.033
CNS infection	26 (2.67%)	22 (2.75%)	4 (2.31%)	0.947
lymphoma	8 (0.82%)	5 (0.63%)	3 (1.73%)	0.318
Kaposi’s sarcoma	4 (0.41%)	4 (0.50%)	0 (0.00%)	0.781
Pneumothorax	20 (2.06%)	7 (0.88%)	13 (7.51%)	<0.001
Coinfection				
HBV	51 (5.25%)	45 (5.63%)	6 (3.47%)	0.332
HCV	22 (2.26%)	17 (2.13%)	5 (2.89%)	0.742
Syphilis	161 (16.56%)	129 (16.15%)	32 (18.50%)	0.521

HIV, human immunodeficiency virus; ART, antiretroviral therapy; HR, heart rate RR, respiratory rate; bpm, beats per minute; PCO_2_, partial pressure of carbon dioxide; PO_2_, arterial partial pressure of oxygen; P (A-a)O_2_, alveolar arterial partial oxygen pressure difference; SpO_2_, blood oxygen saturation; WBC, white blood cell; RBC, red blood cell; HGB, hemoglobin; PLT, platelet; L, lymphocyte; N, neutrophils; ALT, alanine aminotransferase; AST, aspartate aminotransferase; TBIL, total bilirubin; DBIL, direct bilirubin; ALB, albumin; BUN, blood urea nitrogen; CER, serum creatinine; CHO, cholesterol; PALB, prealbumin; CHE, cholinesterase; K, Serum potassium; Na, Serum sodium; Cl, Serum chloride; LDH, lactate dehydrogenase; BDG, Fungi (1,3)-β-D-glucan; CRP, C-reactive protein; CMV, cytomegalovirus; CNS, central nervous system; HBV, Hepatitis B Virus; HCV, Hepatitis C Virus. Data are presented as median [interquartile range, (IQR)] or cases (percentage).

We divided the data into training cohort and testing cohort based on the date of patient admission, and used data from January 2012 to December 2019 to develop a prognosis model (training cohort) and data from January 2020 to October 2022 to validate the model (testing cohort). Despite the temporal disconnect, baseline characteristics were basically comparable in both the training cohorts and testing cohorts (**Table 2**), which were consistent with the overall population.

**Table 2:** Characteristics of patients in training and testing cohorts.

Variables	Training cohort (n=788)			Testing cohort (n=184)		
	Survived (n=639)	Died (n=149)	*P*	Survived (n=160)	Died (n=24)	*P*
Demographics						
Age (years)	37.00 (30.00,47.00)	40.00 (34.00,48.00)	0.007	40.00 (30.00,48.00)	49.00 (36.50,60.00)	0.014
Gender (M/F)	595/44	141/8	0.625	154/6	23/1	1.000
Course of disease (d)	30.00 (10.00,50.00)	20.00 (12.00,42.50)	0.940	20.00 (10.00,30.00)	20.00 (8.50,30.00)	0.965
HIV infection time (d)	14.00 (3.00,180.00)	14.00 (2.00,365.00)	0.923	10.00 (2.00,330.00)	9.00 (2.25,60.00)	0.800
Transmission route			0.311			0.052
Homosexual	499 (78.09%)	116 (77.85%)		131 (81.88%)	15 (62.50%)	
Heterosexual	109 (17.06%)	28 (18.79%)		26 (16.25%)	9 (37.50%)	
Blood transfusion	23 (3.60%)	5 (3.36%)		3 (1.88%)	0 (0.00%)	
Intravenous drug	8 (1.25%)	0 (0.00%)		0 (0.00%)	0 (0.00%)	
ART-naive	558 (87.32%)	129 (86.58%)	0.913	124 (77.50%)	19 (79.17%)	1.000
Vital signs						
HR (bpm)	90.00 (80.00,102.00)	98.00 (88.00,110.00)	<0.001	100.00 (88.00,109.50)	102.00 (90.50,120.00)	0.398
RR (bpm)	20.00 (20.00,22.00)	23.00 (20.00,26.00)	<0.001	20.00 (20.00,22.00)	20.00 (20.00,25.75)	0.765
Symptom						
Fever	534 (83.57%)	125 (83.89%)	1.000	128 (80.00%)	18 (75.00%)	0.769
Cough	445 (69.64%)	94 (63.09%)	0.147	124 (77.50%)	19 (79.17%)	1.000
Shortness of breath	451 (70.58%)	114 (76.51%)	0.178	122 (76.25%)	19 (79.17%)	0.955
Laboratory test						
PH	7.43 (7.41,7.45)	7.43 (7.40,7.46)	0.165	7.44 (7.41,7.46)	7.44 (7.42,7.47)	0.634
PCO_2_ (mmHg)	32.15 (28.88,36.10)	30.10 (27.00,35.55)	0.007	29.00 (26.50,31.25)	26.75 (24.48,31.73)	0.111
PO_2_ (mmHg)	75.55 (64.75,86.80)	53.10 (47.23,60.88)	<0.001	78.00 (65.83,89.95)	52.60 (45.48,58.03)	<0.001
P (A-a)O_2_ (mmHg)	34.85 (21.40,45.90)	59.10 (50.00,66.30)	<0.001	36.10 (23.50,50.40)	63.80 (57.98,66.85)	<0.001
SpO_2_ (%)	95.45 (92.40,97.30)	86.00 (78.30,90.80)	<0.001	95.70 (93.30,97.10)	86.80 (82.30,90.13)	<0.001
WBC (×10^9/L)	4.91 (3.56,6.83)	6.29 (4.08,9.17)	<0.001	5.52 (3.83,7.07)	4.41 (2.38,9.05)	0.534
RBC (×10^12/L)	3.88 (3.38,4.30)	3.75 (3.08,4.36)	0.072	4.11 (3.70,4.62)	3.85 (3.05,4.07)	0.002
HGB (g/L)	114.00 (101.00,126.00)	108.00 (90.00,126.50)	0.011	123.00 (107.00,136.00)	112.50 (90.25,117.00)	0.001
PLT (×10^9/L)	216.00 (159.00,282.00)	183.00 (96.50,270.50)	0.001	242.00 (187.25,308.50)	166.50 (115.00,254.25)	0.002
L (×10^9/L)	0.74 (0.48,1.11)	0.41 (0.26,0.75)	<0.001	0.77 (0.46,1.06)	0.43 (0.23,0.77)	0.003
N (×10^9/L)	3.44 (2.24,5.18)	5.15 (3.21,7.87)	<0.001	4.11 (2.61,5.60)	3.69 (1.88,7.68)	0.903
ALT (U/L)	30.00 (17.30,53.40)	31.60 (20.80,47.60)	0.722	26.35 (15.23,52.75)	26.00 (16.28,52.30)	0.622
AST (U/L)	33.30 (23.90,51.70)	43.50 (31.20,67.95)	<0.001	30.65 (21.05,47.68)	40.00 (35.30,119.30)	0.001
TBIL (μmol/L)	7.80 (6.10,10.00)	10.00 (7.25,13.75)	<0.001	7.05 (5.20,9.90)	13.15 (6.73,21.50)	<0.001
DBIL (μmol/L)	2.30 (1.40,3.40)	3.10 (2.00,5.45)	<0.001	3.00 (2.20,4.00)	4.55 (2.83,13.13)	0.002
ALB (g/L)	32.10 (28.90,35.10)	24.30 (21.65,26.20)	<0.001	33.40 (30.20,36.10)	24.35 (20.23,27.30)	<0.001
BUN (mmol/L)	4.12 (3.20,5.29)	5.22 (4.09,6.85)	<0.001	4.07 (3.37,5.68)	5.26 (3.68,6.99)	0.041
CRE (μmol/L)	64.50 (55.60,74.40)	60.60 (47.75,71.35)	0.002	62.00 (52.25,71.28)	58.00 (46.25,96.43)	0.874
CHO (mmol/L)	3.52 (2.96,4.16)	3.21 (2.66,3.94)	0.002	3.53 (2.98,4.11)	3.60 (2.56,4.90)	0.945
PALB (mg/L)	163.70 (110.45,239.30)	100.40 (67.90,148.10)	<0.001	158.75 (117.53,232.33)	108.10 (92.00,158.13)	0.002
CHE (U/L)	5409.00 (4181.50,6693.00)	3357.00 (2405.00,4435.00)	<0.001	5514.00 (4294.50,6778.00)	3286.50 (2244.00,4612.25)	<0.001
K (mmol/L)	3.94 (3.61,4.32)	3.90 (3.55,4.31)	0.550	4.08 (3.66,4.41)	3.88 (3.71,4.82)	0.943
Na (mmol/L)	135.30 (132.50,137.90)	133.30 (129.55,136.20)	<0.001	135.95 (133.30,138.20)	132.95 (125.43,136.60)	0.004
Cl (mmol/L)	99.80 (96.70,102.40)	98.50 (95.25,101.15)	0.001	101.05 (98.73,103.58)	98.85 (93.85,103.70)	0.114
LDH (U/L)	325.85 (246.95,422.53)	527.15 (409.78,715.50)	<0.001	311.50 (235.75,435.75)	500.00 (382.00,847.00)	<0.001
BDG (pg/mL)	104.75 (14.83,309.30)	94.35 (10.00,330.25)	0.374	109.25 (11.38,212.65)	67.20 (20.80,155.10)	0.351
CD4 (cell/μL)	25.00 (10.00,59.00)	10.00 (4.00,24.00)	<0.001	28.00 (10.00,46.75)	8.00 (3.00,29.00)	0.002
CD4/CD8	0.06 (0.03,0.12)	0.04 (0.02,0.09)	0.001	0.06 (0.03,0.11)	0.04 (0.02,0.09)	0.239
CRP (mg/L)	30.00 (10.00,72.45)	62.00 (24.00,113.00)	<0.001	29.90 (12.55,62.85)	73.50 (16.00,116.80)	0.019
Comorbidities						
Bacterial pneumonitis	475 (74.33%)	126 (84.56%)	0.011	120 (75.00%)	22 (91.67%)	0.120
Tuberculosis	178 (27.86%)	53 (35.57%)	0.078	36 (22.50%)	8 (33.33%)	0.366
Candida	269 (42.10%)	62 (41.61%)	0.987	61 (38.13%)	13 (54.17%)	0.204
Cryptococcus	9 (1.41%)	9 (6.04%)	0.002	6 (3.75%)	2 (8.33%)	0.624
Penicillium marneffei	5 (0.78%)	4 (2.68%)	0.124	2 (1.25%)	0 (0.00%)	1.000
CMV	99 (15.49%)	33 (22.15%)	0.066	45 (28.13%)	11 (45.83%)	0.128
CNS infection	14 (2.19%)	4 (2.68%)	0.953	8 (5.00%)	0 (0.00%)	0.560
lymphoma	1 (0.16%)	1 (0.67%)	0.826	4 (2.50%)	2 (8.33%)	0.377
Kaposi’s sarcoma	2 (0.31%)	0 (0.00%)	1.000	2 (1.25%)	0 (0.00%)	1.000
Pneumothorax	5 (0.78%)	11 (7.38%)	<0.001	2 (1.25%)	2 (8.33%)	0.142
Coinfection						
HBV	36 (5.63%)	6 (4.03%)	0.559	9 (5.63%)	0 (0.00%)	0.494
HCV	17 (2.66%)	4 (2.68%)	1.000	0 (0.00%)	1 (4.17%)	0.271
Syphilis	101 (15.81%)	24 (16.11%)	1.000	28 (17.50%)	8 (33.33%)	0.122

HIV, human immunodeficiency virus; ART, antiretroviral therapy; HR, heart rate RR, respiratory rate; bpm, beats per minute; PCO_2_, partial pressure of carbon dioxide; PO_2_, arterial partial pressure of oxygen; P (A-a)O_2_, alveolar arterial partial oxygen pressure difference; SpO_2_, blood oxygen saturation; WBC, white blood cell; RBC, red blood cell; HGB, hemoglobin; PLT, platelet; L, lymphocyte; N, neutrophils; ALT, alanine aminotransferase; AST, aspartate aminotransferase; TBIL, total bilirubin; DBIL, direct bilirubin; ALB, albumin; BUN, blood urea nitrogen; CER, serum creatinine; CHO, cholesterol; PALB, prealbumin; CHE, cholinesterase; K, Serum potassium; Na, Serum sodium; Cl, Serum chloride; LDH, lactate dehydrogenase; BDG, Fungi (1,3)-β-D-glucan; CRP, C-reactive protein; CMV, cytomegalovirus; CNS, central nervous system; HBV, Hepatitis B Virus; HCV, Hepatitis C Virus. Data are presented as median [interquartile range, (IQR)] or cases (percentage).

### 3.3 Variable selection

Variables were screened according to the following steps.

Step 1: 26 variables were removed from the 59 variables through univariate logistic regression analysis. It was found that BUN levels are influenced by age and gender, with BUN levels typically increasing with age. Due to a strong covariance between BUN and Age, BUN was removed. Additionally, P(A-a)O_2_ is not readily detectable in primary care hospitals in China, making it uneasy to obtain and extrapolate, so it was also removed. Furthermore, RBC, DBIL, PALB, and CD4/CD8 were removed due to their similar clinical significance. Therefore, 27 variables were retained through univariate logistic regression analysis combined with clinical significance of indicators.

Step 2: By employing multivariate logistic regression analysis, 9 variables were further screened out from 27 variables. This analysis revealed that PO_2_, LDH, CD4+T lymphocyte count, N, TBIL, ALB, Cl, RR, and cryptococcal infection were independent predictors of the prognosis of HIV/AIDS patients with PCP, as shown in **Table 3**.

**Table 3:** Multivariate logistic regression analysis of the prognosis of HIV/AIDS patients with PCP.

	*B*	*SE*	*Wald*	*OR*	*P* value	95% *CI*
PO_2_	-0.050	0.023	4.711	0.952	0.030	0.901∼0.995
LDH	0.002	0.001	5.088	1.002	0.024	1.000∼1.004
CD4	-0.010	0.005	4.722	0.990	0.030	0.980∼0.999
N	0.909	0.384	5.601	2.482	0.018	1.169∼5.270
TBIL	0.072	0.030	5.967	1.075	0.015	1.014∼1.139
ALB	-0.341	0.048	49.621	0.711	0.000	0.646∼0.782
Cl	-0.096	0.041	5.614	0.908	0.018	0.838∼0.983
RR	0.124	0.044	8.058	1.133	0.005	1.039∼1.234
Cryptococcus	2.261	0.827	7.478	9.588	0.006	1.897∼48.463
Constant	14.790	5.302	7.780	2649700.685	0.005	

PO_2_, arterial partial pressure of oxygen; LDH, lactate dehydrogenase; N, neutrophils; TBIL, total bilirubin; ALB, albumin; Cl, Serum chloride; RR, Respiratory rate.

**Figure 2:**
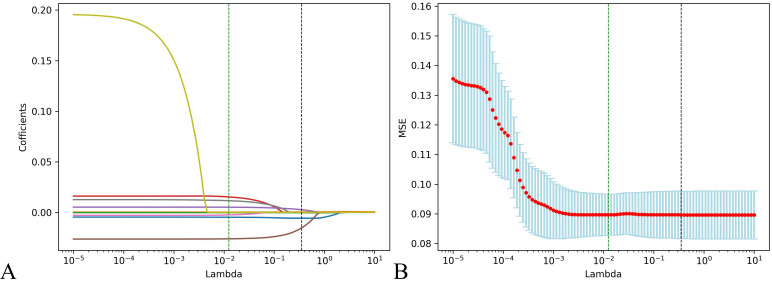
Predictors of the prognosis of HIV/AIDS patients with PCP were selected by using Lasso regression analysis. **(A)** Lasso coefficient profiles of the clinical features; **(B)** The result chart of cross validation of Lasso regression. Identifying the optimal penalization coefficient lambda (λ) through cross-validation, the black dotted line represents the value of the minimum MSE λ Value, in which case the model achieves the best performance.

Step 3: Lasso regression analysis was utilized for precision screening of variables again and to estimate the coefficients of each risk factor in the prognosis of HIV/AIDS patients with PCP. The death outcome during hospitalization was taken as the dependent variable. Nine predictive variables, including PO_2_, LDH, CD4+T lymphocyte count, N, TBIL, ALB, Cl, RR, and cryptococcal infection were included in the Lasso regression model to select predictors related to the prognosis of HIV/AIDS patients with PCP. When the value of λ was 0.351, it indicated that the model performed the best when there are 5 risk factors, as shown in **Figure 2**. ALB, PO_2_, TBIL, LDH, CD4+T lymphocyte count were included in the prediction model.

### 3.4 Establishment and verification of prediction model

**Figure 3:**
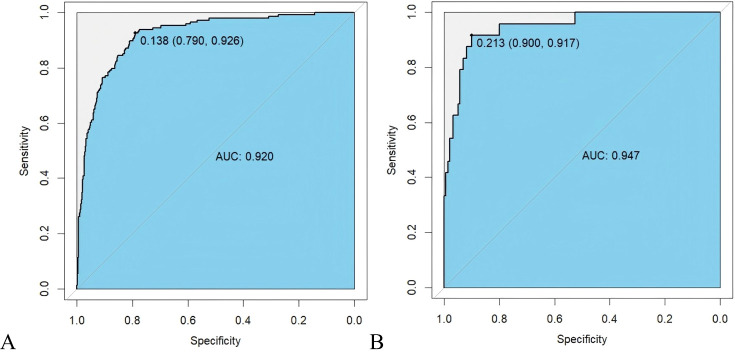
ROC curve of prognosis model of HIV/AIDS patients with PCP. **(A)** ROC curve evaluates discrimination performance in the training dataset with an area under the curve (AUC) of 0.920; **(B)** In the testing dataset, AUC for discrimination performance is 0.947.

The data were divided into training and testing datasets based on the admission time of patients. In the training dataset, the ROC indicated that the resulting model exhibited excellent discrimination with an AUC of 0.994, while the AUC in the testing dataset is 0.947, demonstrating satisfactory discrimination (**Figure 3**). Other model performance evaluation indicators included accuracy, precision, recall, and F1 scores of 0.816, 0.507, 0.926, and 0.656 in the training dataset and 0.902, 0.579, 0.917, and 0.710 in the testing dataset respectively.

**Figure 4:**
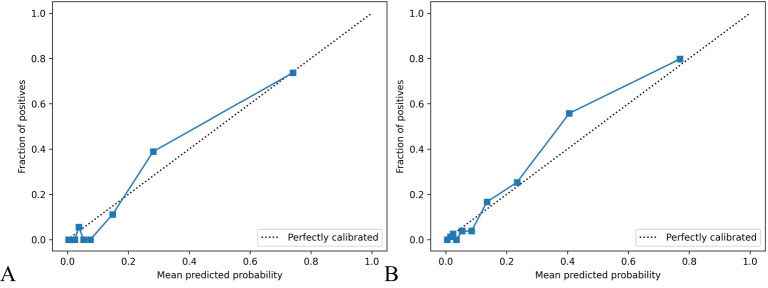
Calibration curve of prognosis model of HIV/AIDS patients with PCP. The calibration curves in **(A, B)** show the consistency of predicting AIDS patients’ prognosis with PCP in both the training and testing datasets. The x-axis represents the model’s prediction probability (ranging from 0 to 1), while the y-axis reflects the actual event rate of patients. The solid blue line represents the actual values corresponding to the predicted values, closely aligning with the diagonal line, indicating good consistency. Hosmer Lemeshow goodness-of-fit test indicates P > 0.05.

**Figure 5:**
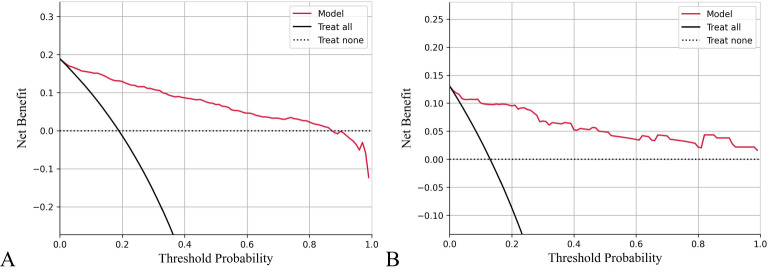
Decision curve analysis (DCA) of prognosis model of HIV/AIDS patients with PCP. The X-axis shows threshold probabilities, and the Y-axis represents net benefit. The red curve depicts the prognosis model for HIV/AIDS patients with PCP. The horizontal dotted line assumes no patient death, while the black diagonal line represents the hypothesis that all patients die. Results indicate that using this prognostic model to predict death risk in HIV/AIDS patients with PCP will yield greater benefits than intervening with all or none of the patients when threshold probabilities range from 0% to 90% for training data **(A)** and 0% to 100% for testing data **(B)**.

The calibration curve for predicting the prognosis of HIV/AIDS patients with PCP showed good consistency between the training and the testing dataset, as depicted in **Figure 4**. The calibration ability of this prognosis model was evaluated with Hosmer-Lemeshow goodness-of-fit test. The results showed that the HL test P value was > 0.05, suggesting that there was no statistically significant difference between the predicted values from the model and actual observed values, indicating good calibration ability. The DCA curve of training dataset and testing dataset is shown in **Figure 5**. When the threshold probability is 0% - 90% and 0% - 100% respectively, it can be observed that more clinical benefits may be brought to HIV/AIDS patients with PCP when making intervention decisions based on the prognosis model.

### 3.5 Visualization of prediction models – interpretability of models

#### 3.5.1 Global interpretability

**Figure 6:**
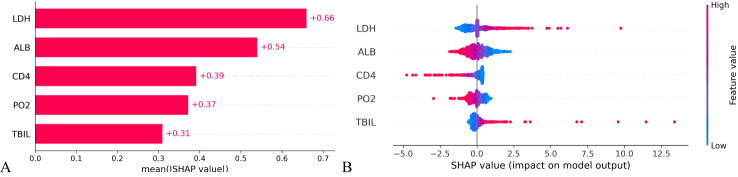
Importance ranking of variable characteristics in the prognosis model of HIV/AIDS patients with PCP. **(A)** Mean SHAP values of the variables indicate the importance of each characteristic in predicting patient mortality. A higher SHAP value corresponds to a greater probability of death prediction. **(B)** The variable SHAP summary plot shows how positive and negative SHAP values affect death prediction for each patient.

The significance of variables is measured through the mean SHAP value, with a higher mean SHAP value indicating a greater impact on the model’s prediction results. Positive or negative SHAP values can promote or reduce the likelihood of death prediction, respectively. Upon ranking the variable importance based on the mean SHAP value, it is evident that LDH holds the highest importance, followed by ALB, CD4+ cell count and PO_2_. Conversely, TBIL is the least influential, see **Figure 6A**. As depicted in **Figure 6B**, larger LDH value (red speckles) correlate with higher corresponding SHAP values, signifying an increased probability of death for patients with elevated LDH levels Similarly, lower levels of ALB, CD4+T cell count and PO_2_ are associated with heightened mortality risk among patients; whereas higher TBIL levels also contribute to increased likelihood of patient mortality.

#### 3.5.2 Local interpretability

**Figure 7:**
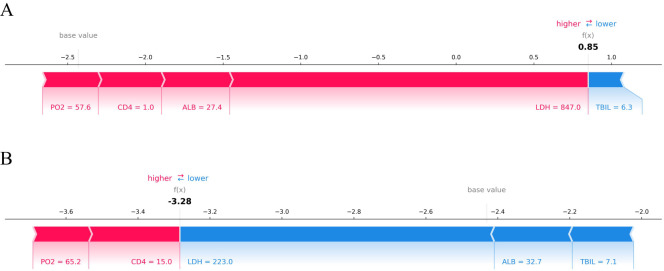
SHAP force plot of HIV/AIDS patients with PCP. The SHAP force plot compares the variable values of a deceased patient **(A)** and a surviving patient **(B)**. Patient **(A)** has a high predicted risk of death with a SHAP value of 0.85, indicated by LDH at 847 U/L, ALB at 27.4 g/L, CD4+ cell count at 1 cell/μL, and PO2 at 57.6 mmHg contributing to an increased risk of death. In contrast, patient **(B)** shows a low predicted risk of death with a SHAP value of -3.28 due to LDH at 223 U/L, ALB at 32.7 g/L, and TBIL at 7.1 μmol/L; these factors are protective prognostic indicators.

**Figure 8:**
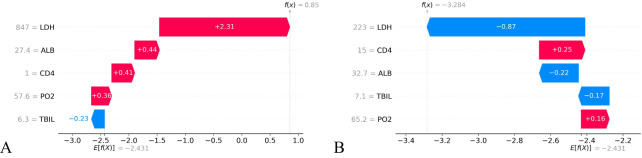
SHAP waterfall plot of HIV/AIDS patients with PCP. The SHAP waterfall plot for a deceased patient **(A)** and a surviving patient **(B)** illustrates the SHAP values. For patient **(A)**, 4 out of 5 features were positive predictions, resulting in a high final SHAP value of 0.85, indicating a greater likelihood of death prediction. Patient **(B)** has two positive predictive characteristics and three negative ones, leading to a lower final SHAP value of -3.284 and decreased probability of death prediction compared to the baseline value.

The SHAP value can effectively clarify and explain the model prediction process for each patient. Randomly select a positive outcome (death patient ID818) and a negative outcome (survival patient ID970) for example. As depicted in **Figures 7** and **8**, different variables contributed differently to the predicted outcomes for patients with different prognoses. Starting from the base value of SHAP (base value, -2.431), four variables (LDH, ALB, CD4 and PO_2_) of the death patient contributed positively to the larger SHAP value and TBIL contributed negatively to the SHAP value, ultimately making the SHAP value for this patient larger (0.85), indicating a higher probability of death prediction. Conversely, the variable for the survival patient contributed more negative SHAP values and consequently resulted in a smaller final SHAP value (-3.28), corresponding to a lower probability of death prediction. A variable with a negative SHAP value indicates a protective effect, suggesting a lower predicted probability of the event (e.g., death).

### 3.6 Development of prognosis risk grade for HIV/AIDS patients with PCP

Finally, a prognosis model scoring system was established. The SHAP values of all variables were weighted and summed according to the linear transformation of the measured value and the SHAP value of each variable. The prognosis score Y was obtained through the expit transformation. The range of Y is 0-100, that is, the prognosis model score is quantified as 0-100. It should be noted that a higher score indicates a worse prognosis for the patients and an increased risk of death.


$$\begin{array}{c} Y\;=\;100\times 1/\{1+exp\;[(0.0051\times LDH\;-\;2.0117)\;+\\ \;(-0.1235\times ALB\;+\;3.8214)\;+\;\\ (-0.011\times CD4\;+\;0.4182)\;+\;\\ (-0.0267\times PO_{2}+\;1.8974)\;+\;\\ \left(0.0754\times TBIL\;-\;0.7057)\;-2.431]\right\} \end{array}$$

$$\begin{array}{c} =\;100/(1\;+\;exp\;(0.0051\times LDH\;-\;0.1235\times ALB\;-\;0.011\\ \times CD4\;-\;0.0267\times PO_{2}+\;0.0754\times TBIL\;+\;0.9886) \end{array}$$

According to the prognosis model score Y and the mortality of HIV/AIDS patients with PCP, the prognosis risk grade of HIV/AIDS patients with PCP was categorized into three grades, namely low-risk, moderate-risk and high-risk. Patients in the low-risk group, with a prognosis score Y ≤ 25, had a mortality rate of 5.0%; patients in the moderate-risk group, with a prognosis score 25 < Y ≤ 50, corresponded to a mortality rate of 45.1%; and patients in the high-risk group, with a prognosis score Y > 50, corresponded to a mortality rate of 80.0%. There is a statistically significant difference in mortality among the three grades (χ^2^ = 419.271, P<0.001). The mortality rate of patients increased gradually with increasing prognosis scores (**Table 4**).

**Table 4:** Prognosis risk grade of HIV/AIDS patient with PCP corresponding to the mortality rate.

Prognosis risk grade	Prognosis score	Died (n)	Survived (n)	Mortality rate (%)
Low-risk group	Y ≤ 25	38	716	5.0
Moderate-risk group	25 < Y ≤ 50	51	62	45.1
High-risk group	Y > 50	84	21	80.0

## 4 Discussion

In this single-center study, we conducted a retrospective analysis of 972 people with HIV/AIDS with a clinical diagnosis of PCP. The overall mortality rate was found to be 17.8%, and five independent and objective predictors of mortality were identified, which were readily available through laboratory tests. Based on these predictors, we developed and validated a prognostic risk scale scoring model for PCP. The results from the validation dataset further demonstrate that the model has good external applicability. In addition, decision curve analysis suggests that using this model assessment can confer greater clinical benefit at a certain risk threshold.

This study found that the case fatality rate of PCP remained at 17.8% in the era of highly effective antiretroviral therapy. This is comparable to the previous 10% to 30% case fatality rate of antiretroviral therapy (Stringer et al., 2002), but in recent years, Huang DY’s research has found that the mortality rate of PCP is still as high as 24.31% (Huang and Lan, 2020) and consistent with the findings of Zhou J (Zhou et al., 2023), indicating that potent antiviral therapy has not significantly improved the prognosis of patients with PCP. Therefore, it is particularly important to screen out objective indicators early on in order to predict the prognostic risk of patients.

Finally, multivariate logistic regression analysis revealed that nine indexes, including PO_2_, LDH, CD4+ T lymphocyte count, N, TBIL, ALB, CL, RR, and cryptococcal infection, were independent predictors affecting the prognosis of patients with HIV/AIDS and PCP. Among them, non-HIV/AIDS-related factors accounted for 7/9, namely PO_2_, LDH, N, TBIL, ALB, CL, RR, indicating that non-HIV/AIDS-related factors may be more accurate than HIV/AIDS-related factors in predicting PCP mortality. This is consistent with previous findings of Matthew W. Fei (Fei et al., 2009).

Utilizing Lasso regression to analyze the aforementioned 9 predictors, we found that the best prediction effect was achieved when ALB, PO_2_, TBIL, LDH, CD4+ T lymphocyte count are incorporated into the PCP risk prediction model. Elevated serum total bilirubin often indicates end-of-organ dysfunction. In addition, bilirubin may also be a marker of sepsis or septic shock, and it has been shown in previous studies to assess disease severity and predict mortality in critically ill and pneumonic patients (Izcovich et al., 2020; Xiao et al., 2022). In addition, age and serum albumin have been reported not only predictors of mortality in patients with PCP, but also predictors of severe PCP (Wang et al., [[NoYear]]; Wang et al., 2020; Feng et al., 2022; Nie, 2023). In this study, age was not included in the final risk prediction model, due to the lower age of the enrolled patients. LDH, although nonspecific, reflects underlying lung inflammation and injury for PCP. Consensus suggests that LDH>500 U/L tends to indicate a poor prognosis (Wang et al., 2021).

This study presents certain advantages over previous studies. First, previous studies have only focused on factors influencing PCP mortality, which has limited the predictive effect of case fatality. In contrast, this study has not only analyzed the impact factors, but also established a prognostic scoring model which significantly improved the diagnostic and predictive ability of PCP mortality rate. Secondly, while imaging can be used to assess the severity of PCP, its subjectivity is strong. The model in this study, however, is based on objective indicators in the blood for risk scoring, which effectively avoided subjective judgmental bias. Finally, the model in this study is more convenient to use, as it quantifies the predicted risk values, allowing clinicians to obtain a determined probability value of PCP mortality through simple calculations within the first 72 hours of the patient’s admission.

The main limitations of this study are the sample source and gender distribution. First, the sample was collected from a single hospital in a specific region, potentially introducing selection bias and limiting generalizability to other areas or healthcare settings. Secondly, 90% of the participants were male, which may reduce the model’s applicability to women. Although no statistically significant difference in mortality rates was observed between genders, there may be indirect effects on patients’ prognosis due to differences in social economic status, access to medical services, and health behaviors, among other factors. To maintain the simplicity and interpretability of the model, we did not include gender as a variable. In addition, our study had begun before “rapid initiation of ART” was widely adopted. As a result, some patients did not start ART even after discharge from PCP (Pneumocystis pneumonia, common in immunocompromised individuals). Historical treatment protocols made it difficult to collect accurate antiviral treatment data for these patients. Therefore, we could not include antiviral therapy status as a variable in our analysis, which we deeply regret.

## 5 Conclusion

Overall, our study found that despite the availability of potent antiviral therapy, mortality in patients with HIV/AIDS and PCP has not decreased in the current era. We developed and validated a prognostic risk model for patients with HIV/AIDS and PCP based on the results of blood tests reviewed by patients at routine visits. While further validation in different regions and medical institutions is necessary, our findings from this study provide a foundation for early identification of mortality risk in PCP patients. Therefore, we also hope that this model can help clinicians assess the severity of the disease and make more precise decisions regarding management strategies.

## Data Availability

The original contributions presented in the study are included in the article/supplementary material. Further inquiries can be directed to the corresponding authors.
